# Effects of video game-based interventions on executive functions and motor skills in children and adolescents with neurodevelopmental disorders: a systematic review and meta-analysis

**DOI:** 10.3389/fresc.2026.1742526

**Published:** 2026-02-27

**Authors:** Ming Gao, Jiabao Ren, Askerbayi Kaulie, Qingjie Wang, Gang Gao

**Affiliations:** 1 School of Physical Education, Xinjiang Normal University, Urumqi, Xinjiang Uygur Autonomous Region, China; 2 Key Laboratory of Special Environment Biodiversity Application and Regulation in Xinjiang, College of Life Sciences, Xinjiang Normal University, Urumqi, Xinjiang Uygur Autonomous Region, China; 3 School of Physical Education and Sports, Soochow University, Suzhou, Jiangsu Province, China

**Keywords:** executive functions, fine motor skills, grossmotor skills, neurodevelopmental disorders, video games

## Abstract

**Purpose:**

This systematic review and meta-analysis aimed to evaluate the effectiveness of video game-based interventions in improving executive functions and motor skills in children and adolescents with neurodevelopmental disorders (NDDs).

**Methods:**

We searched 4 databases: Web of Science, PubMed, Cochrane Library, and IEEE Xplore up to December 2, 2024.

**Results:**

Compared to the control groups, the video game-based intervention groups exhibited a small to medium effect size for inhibitory control (SMD = −0.41, 95% CI: −0.58 to −0.25, *P* < 0.001), cognitive flexibility (SMD = −0.33, 95% CI: −0.50 to −0.15, *P* < 0.001), and working memory (SMD = 0.42, 95% CI: 0.27–0.58, *P* < 0.001) within the domain of executive functions. Additionally, a small to medium effect size was noted in gross motor skills (SMD = 0.45, 95% CI: 0.07–0.82, *P* < 0.05).

**Conclusion:**

Video games can serve as an adjunctive therapy to improve executive functions and gross motor skills in children and adolescents with NDDs. Active video games (AVGs) demonstrate improvements in cognitive flexibility, while sedentary video games (SVGs) show improvements in working memory. Intervention frequency and session duration also influence outcomes. However, due to study heterogeneity and limited sample sizes, these findings remain preliminary and exploratory.

**Systematic Review Registration:**

https://www.crd.york.ac.uk/PROSPERO/view/CRD42024534097, identifier CRD42024534097.

## 1 Introduction

Neurodevelopmental disorders (NDDs) represent a group of conditions arising from atypical development of the nervous system, leading to impairments in multiple functional domains such as cognition, social interaction, communication, emotion, and behavior (1). According to the Diagnostic and Statistical Manual of Mental Disorders, Fifth Edition (DSM-5), NDDs encompass several subtypes, including intellectual disability, communication disorders, autism spectrum disorder (ASD), attention deficit hyperactivity disorder (ADHD), specific learning disorder, and motor disorders [including developmental coordination disorder (DCD), stereotypic movement disorder, and tic disorders]. Among the diverse clinical manifestations of NDDs, executive function deficits serve as a core transdiagnostic impairment (2) and frequently co-occur with motor disorders, forming a stable comorbidity pattern (3, 4). This phenomenon has a neurobiological basis. Neuroimaging studies indicate that the prefrontal-cerebellar-basal ganglia circuitry constitutes an integrated functional network that collectively supports the coordinated development of executive functions and motor skills (5–7). Recent research further confirms a significant positive correlation between executive functions and motor skills (8), providing support for the reciprocity theory. This theory posits that executive functions and motor skills exhibit synergistic promotion during development, with shared neural substrates underpinning bidirectional and reciprocal developmental trajectories (9).

Video games have emerged as a promising intervention tool in the rehabilitation of NDDs in recent years, owing to their capacity to simultaneously integrate physical activity and cognitive tasks (10–12). Compared to traditional interventions, video games offer high levels of immersion and enjoyment, which can effectively enhance participant motivation and treatment adherence. However, current meta-analyses regarding the intervention effects of video games present inconsistent conclusions: some studies support their significant benefits (13–17), while others report limited effect sizes or unstable outcomes (18–23). These discrepancies may stem from variations in critical variables such as participant characteristics, game types, and intervention protocols. Furthermore, although existing meta-analytic evidence supports the positive effects of video games on gross motor skills (24–26), their impact on fine motor skills remains insufficiently explored.

This study aims to systematically evaluate the effects of video game interventions on executive functions and motor skills in children and adolescents with NDDs, with the goal of identifying effective intervention protocols and providing an evidence-based foundation for future research and practice.

## 2 Methods

### 2.1 Registration

This systematic review and meta-analysis followed the Preferred Reporting Items for Systematic Reviews and Meta-Analyses (Z. The protocol of this review was registered on PROSPERO (ID = CRD42024534097).

### 2.2 Study search strategy

We systematically searched four databases—Web of Science, PubMed, Cochrane Library, and IEEE Xplore—from their inception until December 2, 2024. This review only included randomized controlled trials (RCTs) investigating the effects of video games on executive functions or motor skills in children and adolescents with NDDs. The search strategy utilized a combination of subject headings and keywords. This was supplemented by manual searches and reviewing the reference lists of included studies where necessary. Search terms included “video games,” “active video gaming,” “exergaming,” “computer Games,” “autism spectrum disorder,” “attention deficit disorder with hyperactivity,” “motor skills disorders,” “developmental coordination disorder,” “specific learning disorder,” “neurodevelopmental disorders,” “executive function,” “inhibitory control,” “cognitive flexibility,” “working memory,” “inhibition,” “shifting,” “updating,” “cognitive control,” “gross motor skills,” “fine motor skills,” and “motor skills,” etc. Please refer to Supplementary Material 1 for the specific search strategy.

### 2.3 Inclusion and exclusion criteria

This study followed the PICOS framework to define the literature screening criteria, as detailed below: Inclusion criteria: (1) P (Population): Participants must be children and adolescents aged 3–18 years with NDDs diagnosed using either the Diagnostic and Statistical Manual of Mental Disorders (DSM-5 or DSM-IV), the International Statistical Classification of Diseases and Related Health Problems, 10th Revision, or other disorder-specific diagnostic criteria; (2) I (Intervention): Studies employing standalone video game interventions or combined interventions were eligible. For combined interventions, the video game component had to constitute the primary element of the intervention, with the time dedicated to gaming being equal to or greater than that for other components; (3) C (Comparison): The control group should involve no video game intervention or utilize video games that do not exert any influence on the outcome indicators; (4) O (Outcomes): The outcome indicators include executive functions or motor skills; (5) S (Study design): Only RCTs are eligible.

Exclusion criteria: (1) Article published in languages other than Chinese and English; (2) Article with poor quality assessment; (3) Inability to directly or indirectly obtain the mean values and standard deviations of the required outcome indicators.

### 2.4 Data extraction

Following the initial search, MG, JR, and QW screened titles and abstracts. Potentially relevant records then underwent full-text review against the eligibility criteria. JR and QW independently screened articles against eligibility criteria. Discrepancies were resolved through discussion, with MG making final decisions if consensus was not reached. When necessary, AK contacted corresponding authors to request missing data. Studies with irrecoverable incomplete data were excluded.

For studies with multiple outcome measures, we applied a pre-specified prioritization hierarchy to select the primary outcome for extraction, as outlined in the study protocol. This hierarchy was based on the validation status, reliability, and frequency of use in the literature for each NDD population. The highest priority was given to validated and standardized tools with strong psychometric properties in the target population [e.g., Movement Assessment Battery for Children (MABC) for motor skills, Behavior Rating Inventory of Executive Function (BRIEF) for executive functions]; the next priority was assigned to other widely used, validated scales with published reliability and validity data. This pre-specified approach aimed to maximize methodological consistency and minimize measurement heterogeneity across studies.

MG, JR, and QW extracted data using a standardized form, including: (1) study characteristics (author, year, and sample size); (2) demographic characteristics (mean age, diagnosis); (3) intervention details (game name, platform, intervention parameters: duration, session length, frequency); (4) assessment methods. We extracted the mean and standard deviation for the primary outcome measures from both the experimental and control groups at post-test and follow-up time points.

### 2.5 Data analysis

We conducted a meta-analysis of the included continuous variables using Review Manager 5.3 software, calculating the Standardized Mean Differences (SMD) and 95% confidence intervals, with a significance level (*α*) set at 0.05. Effect sizes were categorized as small, medium, and large, corresponding to 0.2, 0.5, and 0.8, respectively (27). Heterogeneity was quantified using the *I*^2^ statistic. In cases where *I*^2^ was less than 50%, the studies were considered homogeneous, and a fixed-effects model was used for analysis; otherwise, a random-effects model was employed. Sensitivity analyses were performed for each indicator-covered study to verify the robustness of the results. To explore potential sources of heterogeneity, subgroup analyses were conducted using a random-effects model.

### 2.6 Bias risk and quality assessment

The quality of the included studies was assessed using the methods recommended by the Cochrane Handbook for Systematic Reviewers, and seven aspects of trial methods that may introduce bias were assessed (sequence generation, allocation concealment, blinding of participants and investigators, evaluators’ blinding, completeness of result data, selective reporting of results, and other biases). As the number of studies included in the meta-analysis for gross motor skills (*n* = 11) exceeded ten, we assessed publication bias for this outcome. Funnel plots were visually inspected, and Egger's test was performed using Stata 16.0 to assess the risk of publication bias.

## 3 Results

### 3.1 Study search results

**Figure 1:**
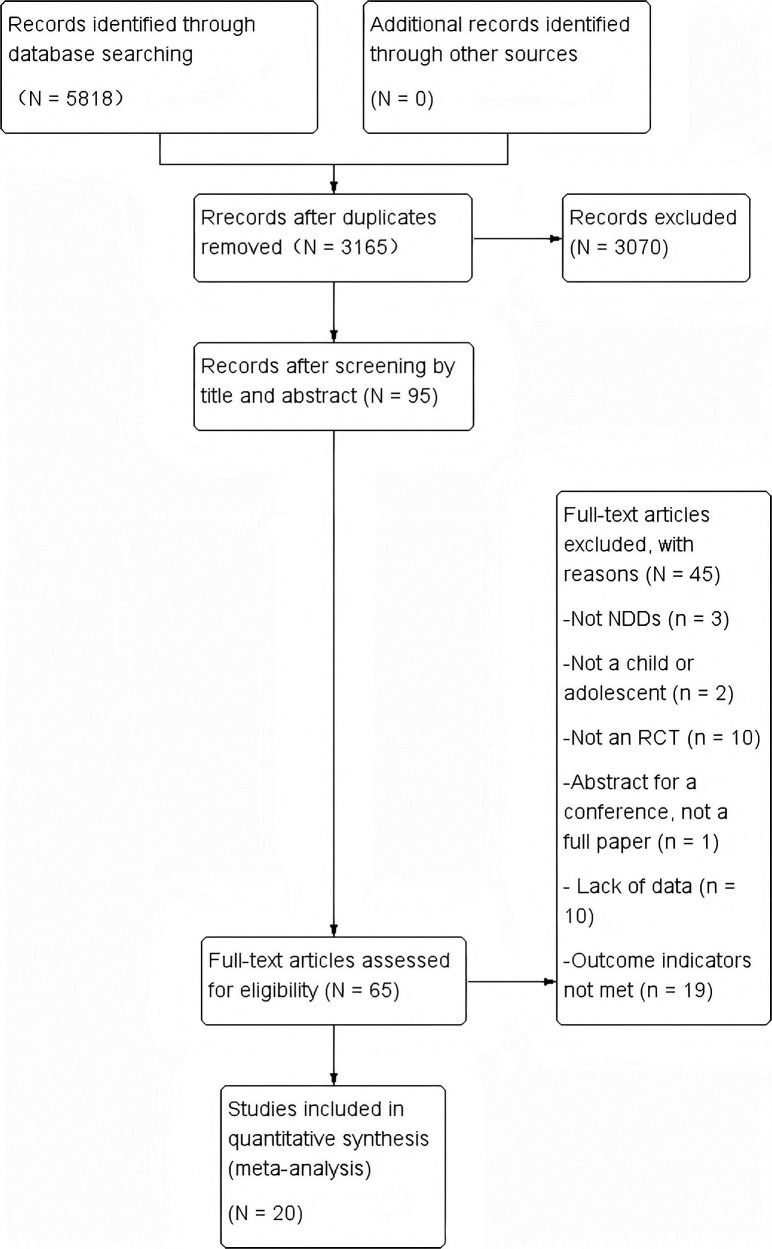
Flow chart of study selection process.

Figure 1 illustrates the screening process of the included studies. During the full-text review stage, the primary reasons for exclusion were missing data (*n* = 10), irrelevant outcomes (*n* = 19), and non-RCTs (*n* = 10, including within-subjects designs (28, 29) and pretest-posttest designs (30–32). After rigorous screening, we ultimately included 20 RCTs.

### 3.2 Characteristics of included studies

The specific characteristics of the included studies are presented in Table 1.

**Table 1:** Characteristics of included studies: participants, intervention protocols, and outcome measures.

Study	Participants	Experimental group			Control group	Intervention duration and frequency	Outcome measures
	Age (Mean ± SD), sample size, and diagnosis	Intervention content	Game types	Characteristics			
Weerdmeester et al. (34)	EG: 9.84 ± 1.71, *N* = 37; CG: 9.69 ± 1.79, *N* = 36; ADHD	Adventurous Dreaming HEGhflying Dragon	AVGs	Full-body game	Active placebo	15 min/session, 2 times/week, 3 weeks	MABC-2, go/no-go task
Nekar et al. (41)	EG: 14.42 ± 5.14, *N* = 12; CG: 14.17 ± 5.09, *N* = 12; ASD	Game contents of UINCARE devices	AVGs	AR + computer-based cognitive training	Cognitive training	15 min/session, 2 times/week, 4 weeks	Stroop Test, WCST, Digit span forward
Bikic et al. (39)	EG: 15.6 ± 0.99, *N* = 9; CG: 9.0, *N* = 8; ADHD	Scientific Brain Training	SVGs	Video games for cognitive training	Active placebo	30 min/session, 5 times/week, 7 weeks	SOC
Bikic et al. (38)	EG: 9.77 ± 1.97, *N* = 35; CG: 10.14 ± 1.52, *N* = 35; ADHD	TAU + Activate	SVGs	Video games for cognitive training	TAU	6 times/week, 8 weeks	SOC, IED, SST
Bul et al. (33)	EG: 9.89 ± 1.28, *N* = 73; CG: 9.82 ± 1.24, *N* = 79; ADHD	TAU + Plan-It commander	SVGs	Serious games to improve daily living function	TAU	65 min/session, 3 times/week, 20 weeks	BRIEF
Dovis et al. (40)	EG: 10.6 ± 1.4, *N* = 31; CG: 10.5 ± 1.3, *N* = 30; ADHD	Brain game brian	SVGs	Video games targeting working memory, inhibition, cognitive flexibility	Active placebo	35–50 min/session, 5 times/week, 5 weeks	Stroop Test, TMT, CBTT
Prins et al. (42)	EG: 9.59 ± 1.12, *N* = 27; CG: 9.33 ± 1.05, *N* = 24; ADHD	Computer game	SVGs	Game elements added to standard computerized WM training	Non-game form of working memory training	35 min/session, 1 time/week, 3 weeks	CBTT
Benzing et al. (43)	EG: 10.46 ± 1.3, *N* = 28; CG: 10.39 ± 1.44, *N* = 23; ADHD	Shape Up	AVGs	Active video games for cognitive function	Waitlist control	30 min/session, 3 times/week, 8 weeks	Simon Task, Flanker task, CBST, German Motor Test
Ji et al. (11)	EG: 9 ± 1.6, *N* = 16; CG: 8.85 ± 1.63, *N* = 14; ADHD	Alchemist's Treasure	AVGs	Running/jumping to dodge obstacles	Bicycle exercise group	50 min/session, 3 times/week, 4 weeks	FAIR
Van Der Oord et al. (44)	EG: 10 ± 0.97, *N* = 18; CG: 9.55 ± 1.43, *N* = 22; ADHD	Brain game Brian	SVGs	Serious game for cognitive rehabilitation	Waitlist control	40 min/session, a total of 25 sessions, 5 weeks	BRIEF
Benzing et al. (35)	EG: 10.46 ± 1.35, *N* = 24; CG: 10.5 ± 1.41, *N* = 22; ADHD	Shape Up	AVGs	Moderate to intense physical activity	Watching documentaries	Moderate to intense intensity for 15 min each time	Flanker Task, CSBT
Milajerdi et al. (12)	EG: 8.15 ± 1.50, *N* = 17; CG: 8.45 ± 1.43, *N* = 20; ASD	Kinect tennis	AVGs	Active video game requiring balance, speed, power, reaction	TAU	35 min/session, 3 times/week, 8 weeks	Mabc-2, WCST
Cavalcante Neto et al. (45)	EG: 8.43 ± 0.81, *N* = 16; CG: 8.12 ± 0.80, *N* = 16; DCD	Will training	AVGs	Upper limb and balance/lower limb skills	Specific task-matching training	60 min/session, 2 times/week, 8 weeks	MABC-2
Ashkenazi et al. (46)	EG: 5.2 ± 0.6, *N* = 15; CG: 15, *N* = 15; DCD	Virtual game	AVGs	Virtual games for upper limb skills	TAU	60 min/session, 10 times/week, 12 weeks	MABC-2
Bonney et al. (36)	EG: 14.3 ± 1.1, *N* = 21; CG: 14.4 ± 1.05, *N* = 22; DCD	Will training	AVGs	VR game with adaptive difficulty	Task-oriented functional training	45 min/session, 1 time/week, 14 weeks	MABC-2
Ju et al. (47)	EG: 6.8 ± 1.3, *N* = 12; CG: 7.0 ± 1.5, *N* = 12; DCD	Imbalance	AVGs	Static and dynamic balance training	No intervention	45 min/session, 3 times/week, 4 weeks	MABC-2
Hammond et al. (48)	EG: 8.5 ± 1.2, *N* = 10; CG: 9.5 ± 1.4, *N* = 8; DCD	Will training	AVGs	Wii Fit games for balance and coordination	TAU	10 min/session, 3 times/week, 4 weeks	BOT-2
Mombarg et al. (49)	EG: 9.5 ± 1.8, *N* = 15; CG: 9.7 ± 1.1, *N* = 14; DCD	Will training	AVGs	Balance training with Wii Balance Board	No intervention	30 min/session, 3 times/week, 6 weeks	MABC-2
Salem et al. (50)	EG: 4.1 ± 0.47, *N* = 20; CG: 4 ± 0.48, *N* = 20; DCD	Will training	AVGs	Balance, strength, walking, aerobic training	TAU	30 min/session, 2 times/week, 10 weeks	GMFM
Vukićević et al. (37)	EG: 10.6 ± 1.52, *N* = 5; CG: 10 ± 1.22, *N* = 5; ASD	TAU +Fruits	AVGs	VR games for static and dynamic motor skills	TAU	10–20 min/session, 1 time/week, 5 weeks	DASH-2

AVGs, active video games; SVGs, sedentary video games; EG, experimental group; CG, control group; TAU, treatment as usual; MABC-2, Movement assessment battery for children-2; SOC, stockings of Cambridge; IED, intra-extra dimensional set shift; SST, stop signal task; BRIEF, behavior rating inventory of executive function; TMT, trail making test; CBTT, corsi block tapping task; FAIR, Frankfurter Aufmerksamkeit-Inventar; CSBT, color span backwards task; WCST, Wisconsin card sorting test; BOT-2, Bruininks-Oseretsky test of motor proficiency-2; GMFM, gross motor function measure; DASH-2, developmental assessment for individuals with severe disabilities-2.

#### 3.2.1 Participants

A total of 878 children and adolescents with NDDs were included as participants in these studies. Among them, the majority were diagnosed with ADHD (*n* = 591, 67.3%), followed by DCD (*n* = 216, 24.6%), and the remainder with ASD (*n* = 71, 8.1%). Studies focusing on executive functions as outcome indicators primarily included participants with ADHD or ASD. Studies with motor skills as the outcome indicator primarily involved participants with DCD.

#### 3.2.2 Study design

Among the 20 included studies, except for one that adopted a crossover randomized controlled trial design (33), the rest all adopted a standard randomized controlled trial design.

#### 3.2.3 Interventions

To improve participants’ executive functions, six articles adopted active video games (AVGs) as interventions, while six employed sedentary video games (SVGs). For the improvement of motor skills, AVGs were universally used. SVGs were conducted on computer platforms, whereas AVG interventions predominantly utilized platforms such as Nintendo Wii and Microsoft Kinect. The intervention cycle ranged from 3 to 14 weeks, with a frequency of 1–10 sessions per week and a single session lasting between 10 and 65 min. However, only a few studies (34–36) mentioned the intensity of the exercise.

#### 3.2.4 Experimental and control groups

Among these studies, except for 3 experimental groups that used video games combined with traditional treatments for intervention (33, 37, 38), the rest only used video games for intervention. The control groups largely did not include video game elements. Although two studies employed digital or commercial games as control interventions (34, 39), these games did not impact the outcome indicators.

#### 3.2.5 Outcome measures

We included 12 studies on executive functions, with 8 reporting on inhibitory control, 7 on cognitive flexibility, and 8 on working memory outcomes. Across these 12 studies, 14 different outcome measures were used, with the most commonly employed tests being Behavior Rating Inventory of Executive Function, Corsi Block Tapping Task, and Stockings of Cambridge. The study results include 3-month follow-up data from Dovis et al. (40), and follow-up results at 12 and 24 weeks from Bikic et al. (38).

Among the 11 included studies on motor skills, the experimental groups all received an AVG intervention. We divided motor skills into gross motor skills and fine motor skills and studied the impact of AVGs on them. Of the 11 studies, 6 involved fine motor skills, and 11 involved gross motor skills. Among them, the most commonly used measurement tools include Movement Assessment Battery for Children-2, Bruininks-Oseretsky Test of Motor Proficiency-2, and Gross Motor Function Measure. For the assessment of gross motor skills, indicators such as balance, bilateral coordination, and jumping were selected; while for the assessment of fine motor skills, manual dexterity test was selected.

### 3.3 Quality assessment and publication bias

**Figure 2:**
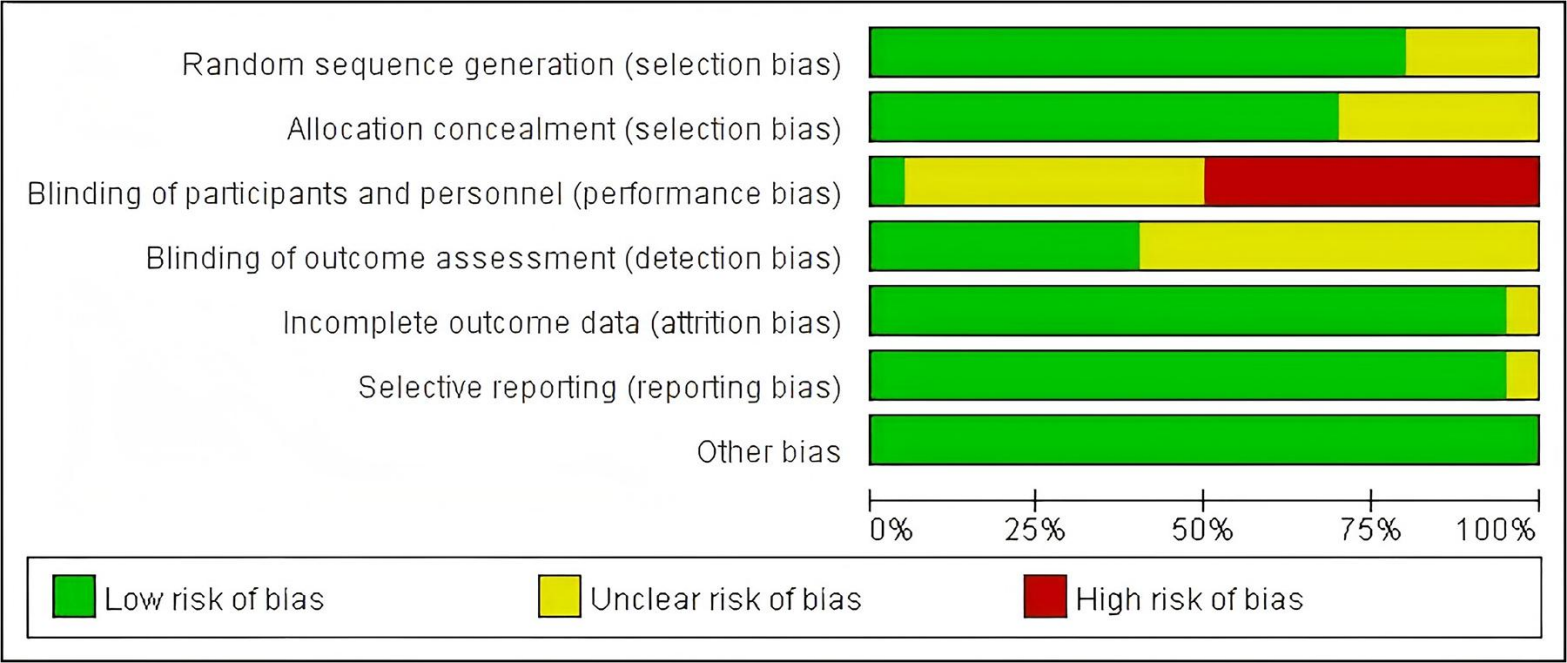
Risk of bias graph presenting an overall assessment of the methodological quality of the included studies.

**Figure 3:**
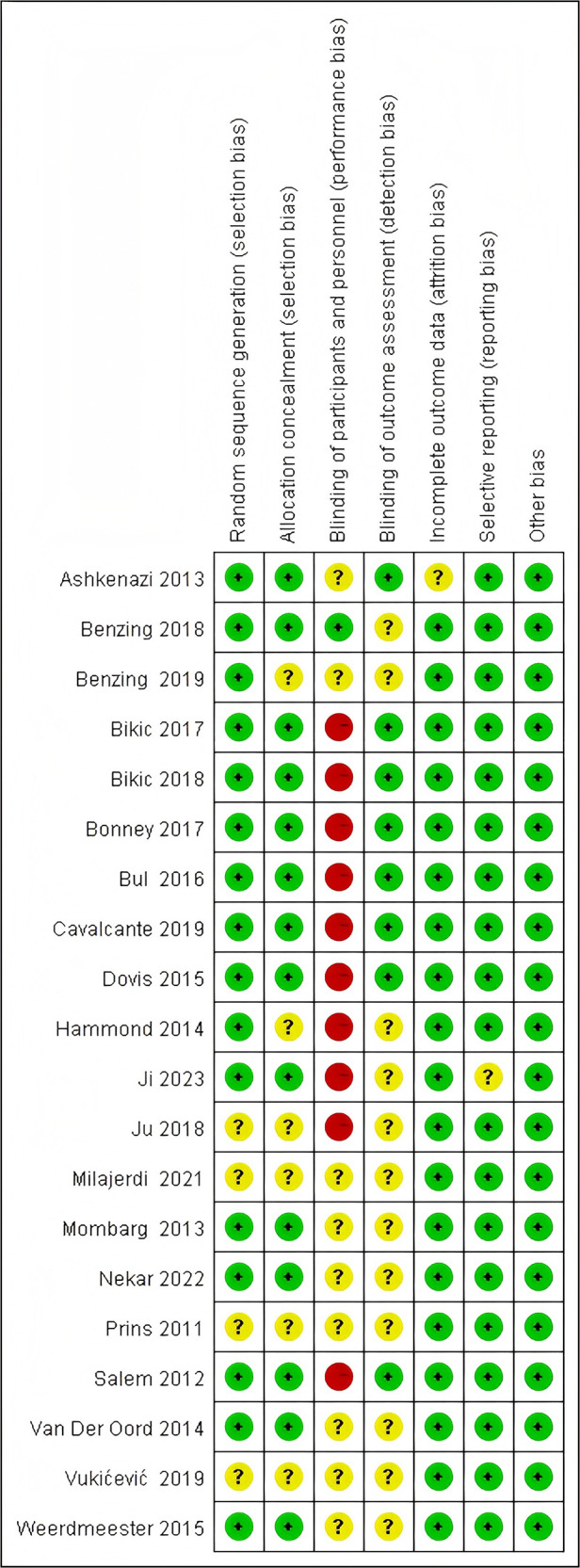
Risk of bias summary showing the distribution of specific methodological limitations across studies.

**Figure 4:**
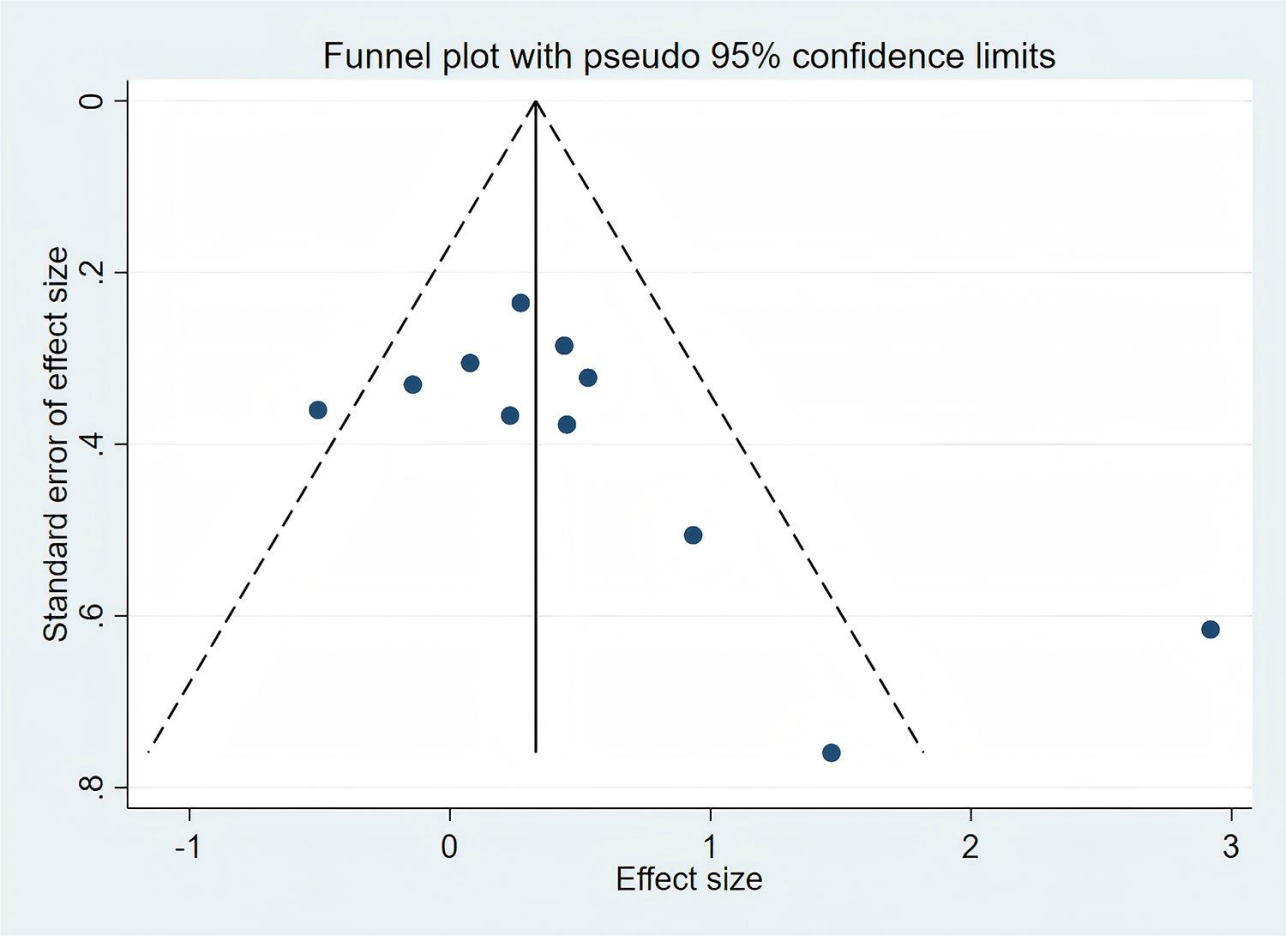
Funnel plot indicating potential publication bias for gross motor skill outcomes. Effect size = standardized mean difference (SMD); each point represents an individual study; asymmetry may indicate potential publication bias.

**Figure 5:**
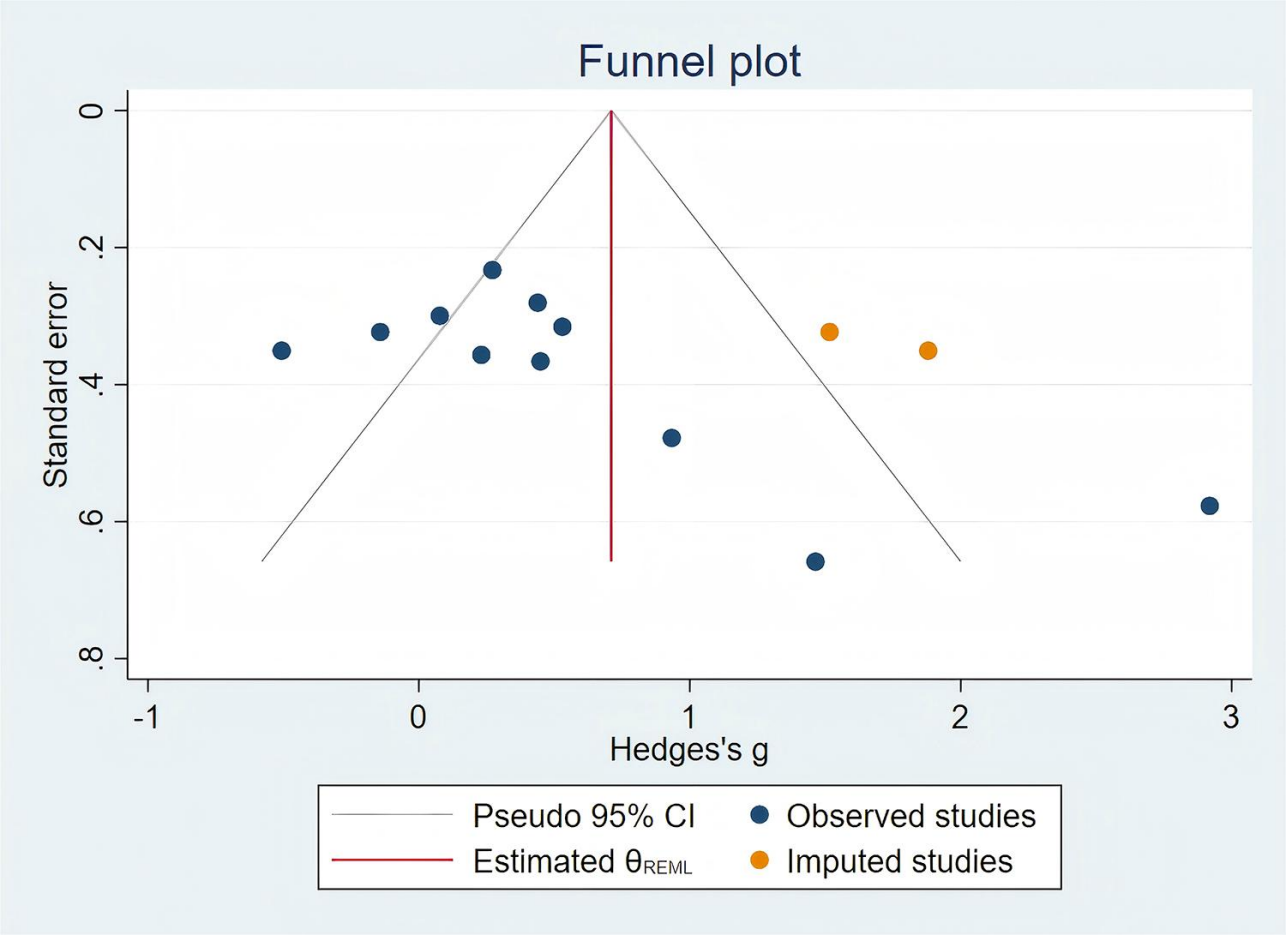
Trim-and-fill analysis suggests adjusted positive effects of video games on gross motor skills. Hedges’ *g* = standardized mean difference corrected for small sample bias. Each blue point represents an included study; orange points indicate imputed studies added by the trim-and-fill method to adjust for potential publication bias. Funnel plot asymmetry suggests potential publication bias.

The results of the literature quality evaluation are presented in Figures 2, 3. Among the 20 included articles, several studies failed to implement double-blinding due to practical difficulties. Figure 4 displays the assessment results of publication bias for studies related to gross motor skills, indicating the presence of publication bias risk (*t* = 2.28; *p* < 0.05; 95% CI: 0.024–7.061). Subsequently, we conducted a trim-and-fill analysis to adjust for this bias, and the outcomes are presented in Figure 5. Following the trim-and-fill analysis, the effect size for the improvement in gross motor skills was adjusted from the original *g* = 0.502 (95% CI: 0.047–0.957) to g = 0.710 (95% CI: 0.234–1.186). This adjustment underscores that despite the presence of publication bias, the positive impact of video game training on gross motor skills remains robust.

### 3.4 Meta-Analysis results

#### 3.4.1 Executive functions

**Figure 6:**
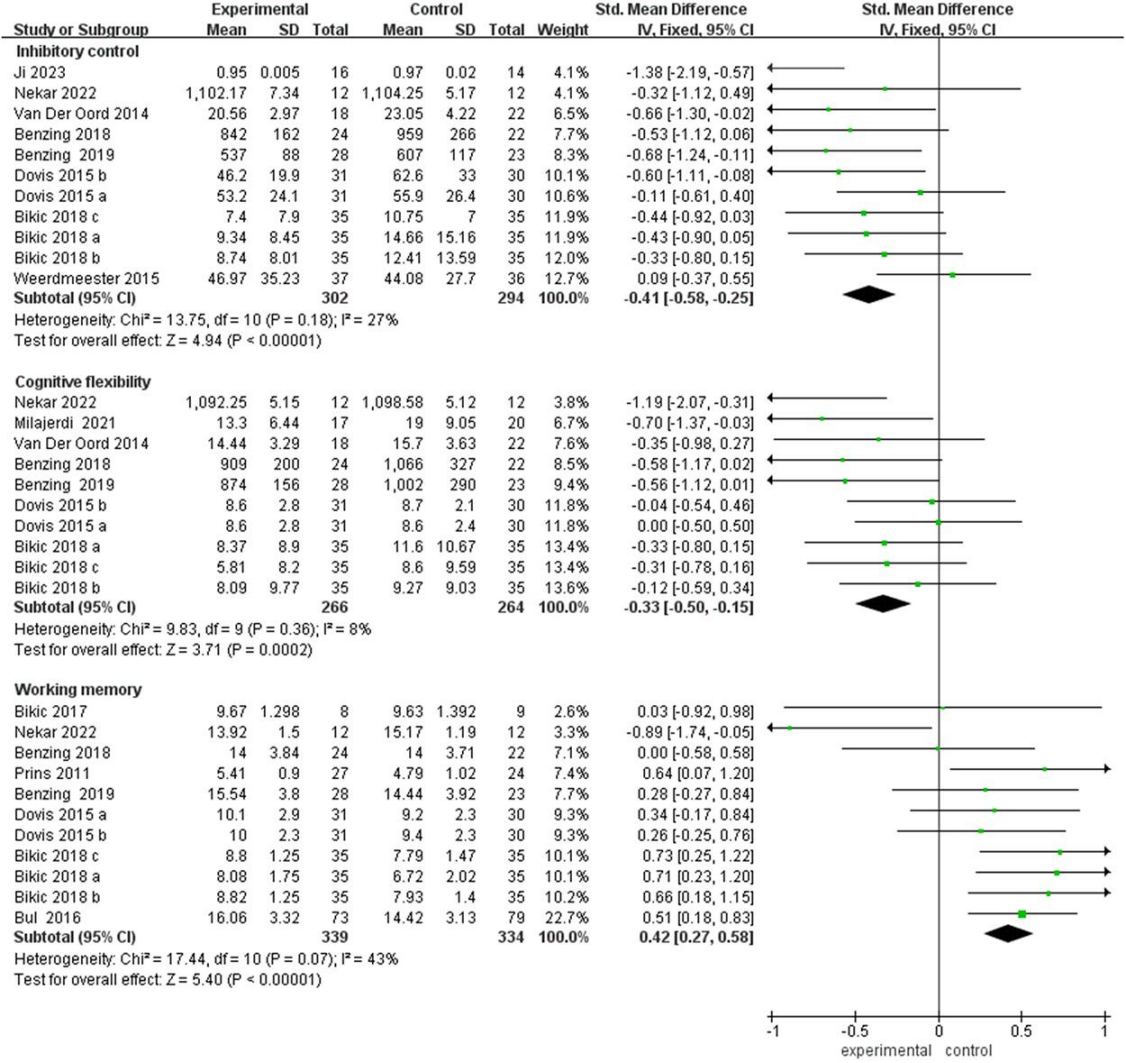
Forest plot showing improvement in executive functions following video game intervention. SMD, standardized mean difference; SE, standard error; CI, confidence interval.

Analysis using a fixed-effects model indicated that the video game intervention yielded significant improvements across all components of executive function: inhibitory control (SMD = –0.41, 95% CI: −0.58 to −0.25), cognitive flexibility (SMD = –0.33, 95% CI: −0.50 to −0.15), and working memory (SMD = 0.42, 95% CI: 0.27–0.58). All *p*-values were <0.001, with effect sizes ranging from small to moderate, as shown in Figure 6. The sensitivity analysis indicates that the results for various cognitive function indicators are stable: inhibitory control (SMD: −0.37 to −0.49, *p* < 0.001, *I*^2^: 0%–35%), cognitive flexibility (SMD: −0.30 to −0.41, *p* < 0.001, *I*^2^: 0%–27%), and working memory (SMD: 0.39–0.47, *p* < 0.001, *I*^2^: 0%–48%) all showed no significant variations.

#### 3.4.2 Motor skills

**Figure 7:**
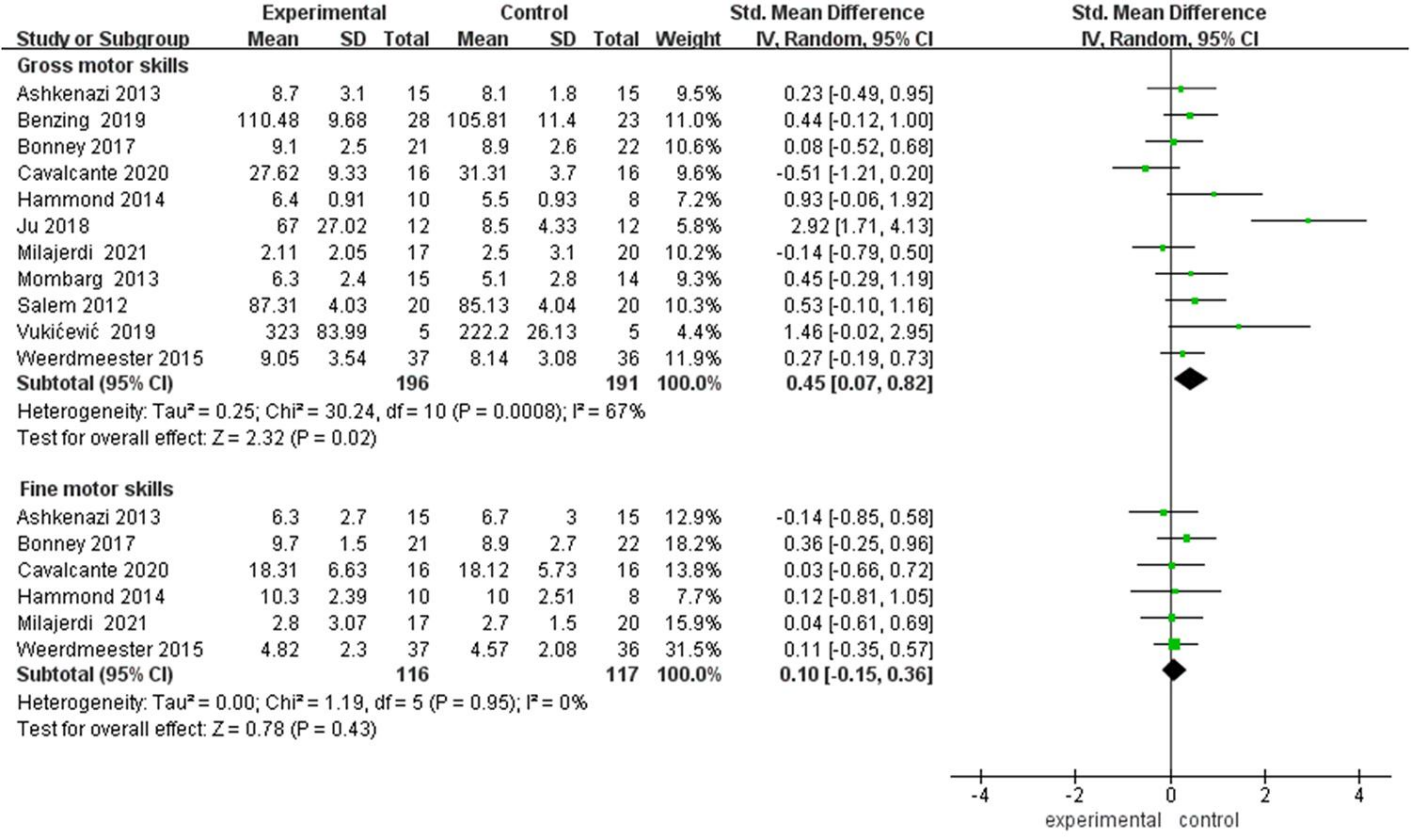
Forest plot of motor skills following video game intervention showing improvement in gross but not fine motor skills. SMD, standardized mean difference; SE, standard error; CI, confidence interval.

Analysis using a random-effects model revealed a small-to-moderate improvement effect of video games on gross motor skills (SMD = 0.45, 95% CI: 0.07–0.82, *p* < 0.05), albeit with substantial heterogeneity (*I*^2^ = 67%), as illustrated in Figure 7. Sensitivity analysis identified the study by Ju et al. (47) as a potential source of heterogeneity; after its exclusion, the effect size decreased to 0.26 (95% CI: 0.01–0.51) and heterogeneity was markedly reduced (*I*^2^ = 25%). Analysis of six studies focusing on fine motor skills did not demonstrate a significant improvement (*p* *>* 0.05). This non-significant result remained unchanged following sensitivity analysis.

#### 3.4.3 Subgroup analysis

To investigate the influence of the intervention protocol and potential sources of heterogeneity, we performed subgroup analyses based on key variables: video game type, intervention duration, session frequency, and session length. The cut-off points for duration, frequency, and session length were determined using median values derived from their respective distributions.

Regarding inhibitory control, both AVGs and SVGs showed small-to-moderate trends of improvement (both *p* < 0.05), with no statistically significant difference between the two groups (*p* *>* 0.05; see Table 2). For cognitive flexibility, AVGs demonstrated a medium-to-large trend of improvement (*p* < 0.001), whereas for working memory, SVGs showed a moderate trend of improvement (*p* < 0.001). Between-group differences for cognitive flexibility and working memory reached statistical significance (*p* < 0.05), though effect sizes and group differences may be influenced by sample size and heterogeneity.

**Table 2:** Subgroup analysis examining the effects of video game intervention on executive functions and motor skills across different moderators.

Indicators	Number of studies	Meta-analysis				*χ* ^2^	*p*
		SMD	95% CI	*I* ^2^	*P* value		
Inhibitory control							
AVGs	5	−0.51	(−0.98, −0.05)	64%	0.030	0.16	0.690
SVGs	3	−0.41	(−0.61, −0.20)	0	<0.001		
>6 weeks	2	−0.45	(−0.70, −0.20)	0	<0.001	0.00	0.940
≤6 weeks	5	−0.43	(−0.82, −0.05)	60%	0.030		
>3 times/week	3	−0.41	(−0.61, −0.20)	0	<0.001	0.13	0.720
≤3 times/week	3	−0.53	(−1.13, 0.08)	73%	0.090		
>30 min	4	−0.42	(−0.67, −0.16)	38%	0.001	0.14	0.710
≤30 min	4	−0.33	(−0.71, 0.05)	40%	0.090		
Cognitive flexibility							
AVGs	4	−0.68	(−1.01, −0.36)	0	<0.001	6.44	0.010
SVGs	3	−0.19	(−0.39, 0.02)	0	0.070		
>6 weeks	3	−0.36	(−0.59, −0.13)	0	0.002	0.07	0.790
≤6 weeks	3	−0.29	(−0.72, 0.14)	51%	0.190		
>3 times/week	3	−0.19	(−0.39, −0.02)	0	0.070	5.88	0.002
≤3 times/week	3	−0.73	(−1.11, −0.34)	0	<0.001		
>30 min	4	−0.23	(−0.43, −0.03)	0	0.020	4.36	0.040
≤30 min	3	−0.68	(−1.05, −0.31)	0	<0.001		
Working memory							
AVGs	3	−0.13	(−0.73, 0.47)	61%	0.070	4.25	0.040
SVGs	5	0.53	(0.36, 0.70)	0	<0.001		
>6 weeks	4	0.55	(0.36, 0.75)	0	<0.001	1.97	0.160
≤6 weeks	3	0.17	(−0.34, 0.67)	66%	0.520		
>3 times/week	3	0.52	(0.31, 0.74)	0	<0.001	1.10	0.290
≤3 times/week	4	0.23	(−0.26, 0.73)	71%	0.020		
>30 min	4	0.54	(0.37, 0.72)	0	<0.001	6.18	0.010
≤30 min	4	−0.08	(−0.55, 0.38)	42%	0.730		
Gross motor skills							
>6 weeks	6	0.13	(−0.17, 0.43)	24%	0.400	4.44	0.040
≤6 weeks	5	1.08	(0.25, 1.91)	78%	0.010		
>3 times/week	1	0.23	(−0.49, 0.95)		0.530	0.30	0.580
≤3 times/week	10	0.46	(0.05, 0.87)	70%	0.03		
>30 min	5	0.39	(−0.42, 1.21)	84%	0.340	0.03	0.860
≤30 min	6	0.47	(0.20, 0.74)	0	<0.001		

AVGs, active video games; SVGs, sedentary video games; SMD, standardized mean difference; CI, confidence interval; *I*^2^, percentage of variation across studies due to heterogeneity; *χ*^2^, chi-squared statistic; *P* value, *p*-value for heterogeneity; “>6 weeks” and “≤6 weeks” indicate intervention duration; “>3 times/week” and “≤3 times/week” indicate intervention frequency; “>30 min” and “≤30 min” indicate session duration.

Analysis of intervention dosage suggested that trends of improvement in cognitive flexibility were mainly observed in subgroups with session lengths ≤30 min (*p* < 0.001) and frequencies ≤3 sessions/week (*p* < 0.001), whereas trends of improvement in working memory appeared more pronounced in subgroups with session lengths >30 min (*p* < 0.001). For gross motor skills, a relatively large trend of improvement was observed only in the subgroup with a total intervention duration ≤6 weeks (*p* < 0.05), though this subgroup showed substantial heterogeneity (*I*^2^ = 78%).

## 4 Discussion

### 4.1 Effects of video games on executive functions and motor skills in children and adolescents with NDDs

Disrupted integration and dynamic instability within the Prefrontal cortex–parietal–subcortical network (involving structures like the caudate nucleus and cerebellum) constitute the core neural basis of EF deficits in children with NDDs. For example, children with ADHD show reduced activation in key nodes of this network (e.g., the inferior frontal gyrus) during task performance, which is directly linked to impairments in response inhibition and WM (51). Children with ASD exhibit abnormalities in prefrontal-cerebellar connectivity and DMN dysfunction, affecting cognitive flexibility (52). Building on this neural mechanism, video game-based interventions can enhance EFs such as attention and inhibitory control by boosting the reward system (e.g., dopaminergic pathways) and promoting plasticity within the aforementioned neural networks (53–55).

Empirical studies show that video game-based interventions outperform control conditions in children and adolescents with ADHD and ASD, a finding supported by systematic reviews. For example, Khan (56) and Ikezawa (57) observed improvements across multiple EF domains in NDDs children in reviews of internet-based interventions including video games. Among 12 related trials, only one reported no significant improvement (38), likely due to low adherence, as only 66.5% of participants completed more than 20 training sessions. Overall, the evidence supports video games as an intervention to improve EF in children with NDDs.

In terms of motor skills, functional deficits in children with NDDs primarily stem from developmental abnormalities in the sensorimotor cortex-cerebellum-basal ganglia circuitry, which impair motor planning, execution, and feedback integration, affecting both gross and fine motor coordination (58, 59). Specifically, children with ASD commonly exhibit cerebellar structural abnormalities and compensatory activation of fronto-striatal circuits (58), whereas children with DCD and ADHD display disrupted interhemispheric connectivity within motor networks, with DCD characterized by a loss of right putamen connectivity dominance (59). These neural alterations form the core basis of motor impairments in NDDs.

Based on this, AVGs exert a positive influence on gross motor skills through full-body motion feedback. Multiple studies support their effectiveness in improving gross motor abilities in children and adolescents. Although neuroimaging evidence specifically for active video games remains limited, other research (e.g., fMRI studies in children with cerebral palsy) indicates that motor training can modulate activation in the fronto-parietal and sensorimotor regions. These neuroplastic changes are closely correlated with behavioral improvements (60). Given that AVG training similarly emphasizes repetition, full-body engagement, and task-oriented practice, it is reasonable to hypothesize that its effects also depend on the plasticity of motor control networks.

In contrast, the efficacy of AVGs in improving fine motor skills appears limited, with only one out of six studies reporting superior performance in hand agility training compared to conventional interventions (36). This limitation may stem from the spatial precision constraints of current mainstream motion-sensing technologies, which hinder their ability to capture fine hand movements with high fidelity. Research indicates that commonly used devices such as Kinect and commercial VR controllers are designed primarily for large-scale limb interactions and exhibit insufficient accuracy in detecting finger-level motions (61, 62). Consequently, these technological limitations likely restrict the potential of AVGs for fine motor training tasks such as grasping and pinching.

In addition to the aforementioned technical limitations of the intervention itself, this study also has certain methodological limitations. The wide age range of participants (3–18 years) increases generalizability but also introduces substantial developmental heterogeneity. According to hierarchical neurodevelopmental theory, basic sensorimotor networks mature relatively early, whereas frontal circuits responsible for higher-order cognitive-motor integration continue developing until late adolescence (63). This aligns with the nonlinear developmental trajectory of the prefrontal cortex: executive functions develop rapidly between middle childhood and mid-adolescence, accompanied by synaptic pruning that optimizes network efficiency and stabilizes by late adolescence (64, 65). Therefore, pooling individuals at different developmental stages in a single analysis may obscure stage-specific intervention effects. Future studies should consider stratification by developmental stage or combine neurodevelopmental features for subgroup analyses to more precisely elucidate intervention outcomes.

Moreover, the majority of the meta-analyses summarized above had small sample sizes. This makes the pooled estimates particularly susceptible to small-study effects and potential publication bias, meaning the observed effect sizes may be inflated. Therefore, while promising, these results should be considered preliminary and require confirmation in larger, more robustly designed trials.

### 4.2 Subgroup analyses

Given the substantial heterogeneity among the included studies in terms of diagnosis, intervention protocols, and other factors, this subgroup analysis aimed to explore the influence of key variables (such as video game type, dosage, and frequency) on effect size variability, rather than to identify a single “optimal” protocol. It should be emphasized that this analysis was an exploratory *post-hoc* assessment; each subgroup included only 3–6 studies and involved multiple comparisons, resulting in limited statistical power and an increased risk of false-positive findings. Therefore, the results should be interpreted with caution.

Specifically, for cognitive flexibility, subgroup analyses based on game type, intervention frequency, and session duration generally reduced within-group heterogeneity, with AVGs appearing to yield more pronounced improvements than SVGs. This indicates that this outcome may show a certain degree of consistency across studies, and its effect size could be influenced by intervention characteristics. This may be because AVGs, by integrating physical movement with cognitive tasks, co-activate the sensorimotor cortex, prefrontal executive control network, and cerebellar–basal ganglia circuitry. Such multi-system engagement is particularly beneficial for cognitive flexibility, which depends on the dynamic interaction between the dorsolateral prefrontal cortex and the dorsal anterior cingulate cortex (66). Moreover, the high metabolic demands of the prefrontal cortex may explain why lower-dose AVG interventions (≤3 sessions/week, ≤30 min/session) still produced considerable effects in our pooled data.

In contrast, inhibitory control exhibited moderate overall heterogeneity and was insensitive to the examined dosage and format variables, with within-subgroup heterogeneity not systematically decreasing. This may reflect its neural substrate, which is primarily governed by the structurally stable right inferior frontal gyrus, conferring relatively limited plasticity in response to variations in training parameters (67).

The analysis of working memory and gross motor skills revealed more complex patterns. For working memory, subgroup analysis indicated that SVGs and longer single-session durations (>30 min/session) were associated with improved outcomes, aligning with the mechanism whereby SVGs primarily engage the dorsolateral prefrontal–parietal working memory network and rely on deep, sustained engagement in processes such as rule memorization and strategic planning (68, 69). For gross motor skills, short-term interventions (≤6 weeks) showed higher effect sizes, possibly reflecting the rapid progression phase in the early stages of motor skill acquisition.

However, residual within-subgroup heterogeneity remained for both outcomes, suggesting that effect variability may also be influenced by other methodological and contextual factors, such as differences in sample characteristics, inconsistencies in measurement tools, and specific details of intervention implementation. Therefore, a single clinical variable is insufficient to fully account for these effect patterns, and future research should further control for these potential sources of variability.

In summary, for outcomes with relatively high consistency, such as cognitive flexibility, the findings provide limited yet meaningful support for cross-diagnostic data pooling under a random-effects model. However, substantial residual heterogeneity remains for outcomes such as working memory and gross motor skills, making it difficult to directly generalize the pooled effects to specific diagnostic subtypes or intervention protocols. Therefore, the pooled estimates presented in this report should be interpreted as reflecting overall trends rather than serving as direct clinical prescriptive recommendations.

### 4.3 Limitations and future directions

Several limitations of this study warrant consideration when interpreting the results: 1) a limited number of included studies with generally small sample sizes, and potential publication bias across all outcome domains; 2) the heterogeneity in diagnoses and intervention protocols among the included studies limits the specificity and direct clinical generalizability of the pooled results; 3) absence of data on the critical variable of exercise intensity; 4) a notable lack of long-term follow-up data, which prevents the assessment of effect sustainability and stability; 5) Due to insufficient data, stratified analyses based on specific NDD subtypes and developmental stages could not be conducted. Future research should focus on standardizing intervention dosage, enhancing intensity monitoring, expanding sample sizes, employing more consistent assessment tools, and prioritizing the collection of long-term follow-up data. This will allow for a deeper investigation into the persistence and stability of intervention effects, as well as their transfer to real-world situations and related cognitive tasks.

## 5 Conclusion

Video games represent a promising adjunctive intervention; however, their clinical application still requires confirmation through higher-quality evidence. Current findings indicate that intervention effects are modulated by key parameters such as game type and dosage, providing preliminary support for the development of more targeted and individualized intervention strategies. In contrast, constrained by the precision of current interaction and motion-capture technologies, video games show limited efficacy in improving fine motor skills, and their intervention potential warrants further evaluation alongside advances in related technologies.

## Data Availability

The original contributions presented in the study are included in the article/Supplementary Material, further inquiries can be directed to the corresponding author.
