# Supplementary material for: Effects of video game-based interventions on executive functions and motor skills in children and adolescents with neurodevelopmental disorders: a systematic review and meta-analysis
Source: Front Rehabil Sci. 2026 Feb 27;7:1742526. doi: 10.3389/fresc.2026.1742526 (PMC12982429; doi:10.3389/fresc.2026.1742526)
Supplement: Supplementary file 1 [file Table1.docx]

| #1 | video game* OR serious game* OR exergam* OR computer game* OR web-based OR web-supported OR computer-based OR computer-supported OR active video game* OR Virtual Reality OR Kinect xbox OR Wii sport OR Wii Fit OR Kinect Sport OR virtual reality OR educational virtual realit* OR instructional virtual realit* OR kinect OR wii sport OR wii fit OR nintendo wii |
| --- | --- |
| #2 | cognition* OR cognitive function* OR cognitive benefits OR cognitive performance OR inhibitory control OR inhibition OR response inhibition OR effort control OR cognitive control OR working memory OR memory OR short-Term memor* OR immediate memor* OR shortterm memor* OR Immediate Recall* OR decision making OR shifting OR cognitive flexibility OR shift cognitive OR updating OR motor skill* OR gross motor skill* OR fine motor skill* OR object control OR manipulation control OR balance |
| #3 | Attention Deficit Hyperactivity Disorder* OR ADHD OR Attention Deficit Disorder with Hyperactivity OR Attention Deficit Disorders with Hyperactivity OR Attention Deficit-Hyperactivity Disorder* OR Attention Deficit Disorder* OR Hyperkinetic Syndrome OR Autism Spectrum Disorder* OR ASD OR Autistic Spectrum Disorder* OR Autistic Disorder OR Autism OR Specific Learning Disorder* OR Learning Disorder* OR Speech Sound Disorder OR Communication Disorders OR Language Disorder OR Motor Disorders OR Developmental Coordination Disorder* OR Motor Skills Disorder* OR Tic Disorder* OR Neurodevelopmental Disorder* OR DCD OR NDDs or Other Neurodevelopmental Disorder* |
| #4 | child* OR school age OR youth preschool OR preschoolers OR adolescen* OR teenage* OR youth OR student* |
| #5 | 1 AND 2 AND 3 AND 4 |

**Search strategy for Pubmed and Cochrane Library**

**Search strategy for Web of Science**

| #1 | TS=(video game* OR serious game* OR exergam* OR computer game* OR web-based OR web-supported OR computer-based OR computer-supported OR active video game* OR Virtual Reality OR Kinect xbox OR Wii sport OR Wii Fit OR Kinect Sport OR virtual reality OR educational virtual realit* OR instructional virtual realit* OR kinect OR wii sport OR wii fit OR nintendo wii) |
| --- | --- |
| #2 | TS=(cognition* OR cognitive function* OR cognitive benefits OR cognitive performance OR inhibitory control OR inhibition OR response inhibition OR effort control OR cognitive control OR working memory OR memory OR short-Term memor* OR immediate memor* OR shortterm memor* OR Immediate Recall* OR decision making OR shifting OR cognitive flexibility OR shift cognitive OR updating OR motor skill* OR gross motor skill* OR fine motor skill* OR object control OR manipulation control OR balance) |
| #3 | TS=(Attention Deficit Hyperactivity Disorder* OR ADHD OR Attention Deficit Disorder with Hyperactivity OR Attention Deficit Disorders with Hyperactivity OR Attention Deficit-Hyperactivity Disorder* OR Attention Deficit Disorder* OR Hyperkinetic Syndrome OR Autism Spectrum Disorder* OR ASD OR Autistic Spectrum Disorder* OR Autistic Disorder OR Autism OR Specific Learning Disorder* OR Learning Disorder* OR Speech Sound Disorder OR Communication Disorders OR Language Disorder OR Motor Disorders OR Developmental Coordination Disorder* OR Motor Skills Disorder* OR Tic Disorder* OR Neurodevelopmental Disorder* OR DCD OR NDDs or Other Neurodevelopmental Disorder* ) |
| #4 | TS=(child* OR school age OR youth preschool OR preschoolers OR adolescen* OR teenage* OR youth OR student*) |
| #5 | 1 AND 2 AND 3 AND 4 |

**Search strategy for iEEE Xplore**

| #1 | video game* OR serious game* OR exergam* OR computer game* OR web-based OR web-supported OR computer-based OR computer-supported OR active video game* OR Virtual Reality OR Kinect xbox OR Wii sport OR Wii Fit OR Kinect Sport |
| --- | --- |
| #2 | cognition* OR cognitive function* OR cognitive benefits OR cognitive performance OR inhibitory control OR inhibition OR response inhibition OR working memory OR memory OR shifting OR cognitive flexibility OR cognitive control OR shift cognitive OR updating OR motor skill* OR gross motor skill* OR fine motor skill* OR object control OR manipulation control OR balance |
| #3 | Attention Deficit Hyperactivity Disorder* OR Attention Deficit Disorder with Hyperactivity OR Attention Deficit-Hyperactivity Disorder* OR Autism Spectrum Disorder* OR Autistic Disorder OR Autism OR Specific Learning Disorder* OR Learning Disorder* OR Motor Disorders OR Developmental Coordination Disorder* OR Motor Skills Disorder* OR Tic Disorder* OR Neurodevelopmental Disorder* |
| #4 | child* OR school age OR youth preschool OR preschoolers OR adolescen* OR teenage* OR youth OR student* |
| #5 | 1 AND 2 AND 3 AND 4 |
